# Piloting a systems level intervention to improve cervical cancer screening, treatment and follow up in Kenya

**DOI:** 10.3389/fmed.2022.930462

**Published:** 2022-09-15

**Authors:** Natabhona M. Mabachi, Catherine Wexler, Harshdeep Acharya, May Maloba, Kevin Oyowe, Kathy Goggin, Sarah Finocchario-Kessler

**Affiliations:** ^1^Practice-Based Research, Innovation, and Evaluation Division, American Academy of Family Physicians, Leawood, KS, United States; ^2^Department of Family and Community Medicine, University of Kansas Medical Center, Kansas City, KS, United States; ^3^Global Health Innovations, Nairobi, Kenya; ^4^Children’s Mercy Research Institute, Kansas City, MO, United States

**Keywords:** eHealth (mobile Health), system—level, cervical cancer screening, cervical cancer treatment, Kenya, cervical cancer tracking

## Abstract

Although preventable, Cervical Cancer (CC) is the leading cause of cancer deaths among women in Sub-Saharan Africa with the highest incidence in East Africa. Kenyan guidelines recommend an immediate screen and treat approach using either Pap smear or visual screening methods. However, system (e.g., inadequate infrastructure, weak treatment, referral and tracking systems) and patient (e.g., stigma, limited accessibility, finance) barriers to comprehensive country wide screening continue to exist creating gaps in the pathways of care. These gaps result in low rates of eligible women being screened for CC and a high loss to follow up rate for treatment. The long-term goal of 70% CC screening and treatment coverage can partly be achieved by leveraging electronic health (eHealth, defined here as systems using Internet, computer, or mobile applications to support the provision of health services) to support service efficiency and client retention. To help address system level barriers to CC screening treatment and follow up, our team developed an eHealth tool—the Cancer Tracking System (CATSystem), to support CC screening, treatment, and on-site and external referrals for reproductive age women in Kenya. Preliminary data showed a higher proportion of women enrolled in the CATSystem receiving clinically adequate (patients tested positive were treated or rescreened to confirm negative within 3 months) follow up after a positive/suspicious screening, compared to women in the retrospective arm.

## Introduction

Cervical Cancer (CC) is preventable through HPV vaccination and screening to detect precancerous lesions; yet in 2018, an estimated 570,000 cases were diagnosed and 311,000 deaths occurred globally (1). In Kenya, CC is the second leading cancer in women, and the leading cancer-related death in women. Around 5,250 new cases of CC are diagnosed annually, and 3,200 women die of CC every year in Kenya (2).

Early detection and treatment of abnormalities and precancers that are likely to progress to invasive carcinoma (3) not only improves overall patient outcomes (5 year survival is 95.7 vs. 9.9% for CC diagnosed at stage 1a1 vs. stage IVb (4, 5) but also reduces the cost of treatment to the patient and health system (USD $85 for stage 0 vs. $1575 for stage III curative) (6). In most cases, CC is due to human papillomavirus infection (7). When HPV infection persists, the time from initial infection to development of high-grade cervical intraepithelial neoplasia and, finally, invasive cancer takes an average of 15 years. This combined with the system and patient screening barriers experienced in resource limited settings highlights the importance of frequent screening to detect intraepithelial disease, for prompt management. To achieve population level health gains, the WHO recommends countries reach 70% coverage with screening and 90% of those needing it receiving treatment (8).

Although awareness of the importance of CC screening is increasing (9), only 16–19% of eligible Kenyan women 18–69 years old are screened for CC (10). These low rates of screening contribute to 50–80% of cases identified in advanced stages of CC (11–13). Due to low cost, Visual inspection with acetic acid (VIA) and Visual inspection with Lugol’s iodine (VILI) techniques are the most used screening methods in a low resource settings like Kenya. Kenyan guidelines call for a “screen and treat” protocol for women with pre-cancerous lesions identified by VIA/VILI (when PAP or HPV testing is unavailable), in which treatment is administered on the same clinic visit as diagnosis to minimize patient burden, treatment delay, and loss to follow-up (LTFU) (14). However, in a survey of 12 hospitals across 7 counties, only 25% of facilities had a functional cryotherapy machine (9), allowing them to offer services compliant with these guidelines. Even amongst those who offer same day services, supply stock outs, C02 shortages for the cryotherapy machine, and lack of trained providers available to perform the procedure can delay treatment and cause increased burden on patients (15, 16). Of those who do not receive same day services, only 20–53% of those diagnosed returned for cryotherapy (15, 16). Indeed, in a multinational study in sub-Saharan African capitals, only 15.8% of CC patients received care with curative potential (17). Individual and social barriers to treatment include concerns about side effects, treatment−related fear and stigma, fear of marital discord, religious and cultural beliefs, geographical location (rural vs. urban), and limited knowledge (18, 19). Financial constraints and long distances to travel for available services also pose substantial challenges to treatment access (20).

eHealth and eHealth interventions have been widely implemented in the past decade to support a range of health-related outcomes and have the potential to improve CC screening and follow up care. In Kenya, eHealth interventions have increased maternal retention in guideline-adherent PMTCT services and on-time testing of infants exposed to HIV (21, 22), and have improved postoperative circumcision follow up (23). In Kenya, patients who received CC rescreening reminders *via* SMS were eight times more likely to adhere to scheduled rescreening than those who did not (24). In a study evaluating the impact of sending CC screening results *via* SMS or phone call, women found both options acceptable and effective and resulted in higher treatment follow up than relying on patient clinic visits to relay results (25). Adapting proven eHealth strategies offers countries low cost strategies to address persistent gaps in the continuum of CC care.

## Materials and methods

This study presents the findings from a pilot study utilizing an eHealth tool—the Cancer Tracking System (CATSystem), an adaptation of a web-based eHealth intervention called the HIV Infant Tracking System (HITSystem) (21). Designed for use in low to middle income settings, the primary goals of the CATSystem are to (a) increase rates of CC screening, (b) improve the treatment, referral (internal and external), and follow up rates of women screened positive with precancerous and cancerous lesions and c) identify missed re-screening and treatment opportunities. Using algorithm driven alerts for providers and SMS to patients, the CATSystem is designed to support CC screening, treatment, and referrals for reproductive age women (HIV + and HIV–) in Kenya. The system accesses satellite broadband *via* modems, generates a provider dashboard linking to patients who are overdue for a service or in need of patient outreach, and sends automated customized text messages to women to support screening and treatment follow-up per national Kenyan Ministry of Health guidelines (14). Decentralized for data entry, authorized providers (mentor mothers, data clerks, or clinicians) enter data in real-time at implementing hospitals, allowing the generation of timely alerts and provider follow up (patient tracing, phone calls, or SMS); however, as a web-based intervention, centralized updates to the programming of the system are automatically applied across sites as they become available.

### Study overview

This study was an observational study with historical controls to evaluate an 11-month pilot of the CATSystem at one provincial level hospital in Rift Valley, Kenya. The standard of care at the facility includes paper-based record keeping for CC screening (VILLI/VIA, pap smear and colposcopy) and on-site treatment with cryotherapy and loop electrosurgical excision procedure (LEEP). Patients needing chemotherapy or radiation treatment are referred to Kenyatta National Hospital, in Nairobi, which is approximately 160 km (3–4 h drive one-way) from the study hospital.

### Cancer tracking system intervention

**FIGURE 1:**
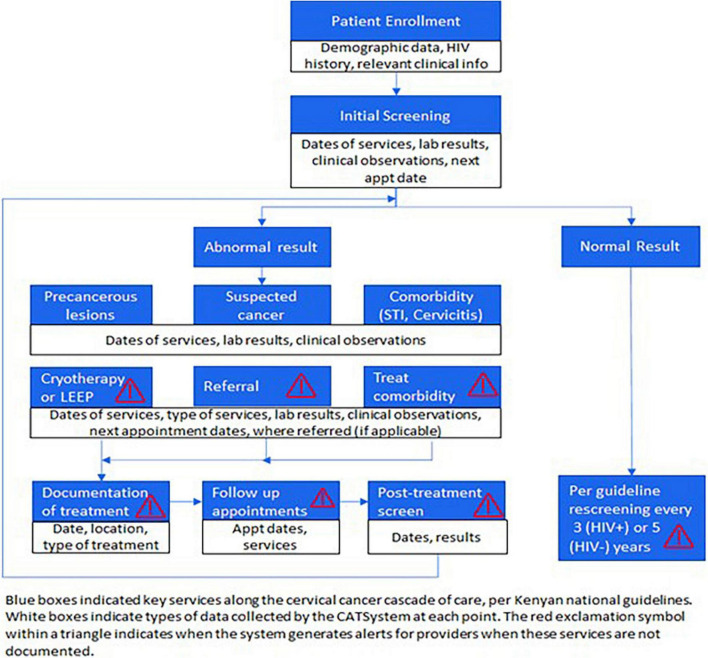
Kenya ministry of health national cervical cancer screening guidelines.

Adapted from the HITSystem, which is an eHealth intervention that has proven effective in improving maternal and infant HIV care in Kenya (21, 22), the CATSystem is an eHealth intervention that aims to improve follow up after an abnormal CC screen and increase rescreening rates per Kenya Ministry of health Guidelines (see Figure 1). Women are enrolled in the CATSystem through the comprehensive HIV care centers (CCC) and maternal and child health/Family Planning (MCH/FP) department. Demographic and contact information are captured at the time of enrollment. The patient is assessed for risk factors of CC including HIV status, age of onset of sexual activity, number of sexual partners, history of sexually transmitted infections, prior positive screenings, or history of *in situ* carcinoma of the vulvar or vaginal epithelium. Women are screened and those who have an abnormal CC screen are ideally treated on the same day. If the patient is unable to undergo same day treatment after a positive screen, the system sends an automated SMS alerts prior to the scheduled appointment and alerts in case of a missed appointment. They are tracked until they complete clinically indicated care based on their unique clinical presentation. After completing appropriate management for suspicious malignancy/*in situ* carcinoma or invasive malignancy, the patient continues to get automated alerts for follow up care based on national guidelines. Women who have a normal screen are prompted for rescreening at the indicated interval. Algorithm-driven electronic alerts notify clinical providers when patients are overdue or missing key services (treatment, labs, follow up care, missed appointments). The system also keeps a record of clinical findings, visual images of the cervix and lab results from each encounter.

The CATSystem was piloted in the Comprehensive Care Center (CCC, where HIV services are provided) and Maternal and Child Health Departments (MCH) from October 22nd, 2019, to January 26th, 2021. These departments are the two main points where CC screening occurs. The inclusion of the CCC also allowed us recruit women living with HIV who are vulnerable to CC due to the associated risk between HIV and contracting the Human Papilloma Virus (HPV) (26, 27). Each department had one or two nurses or clinical officers to conduct screenings and cryotherapy treatments. The facility had two gyno-oncologists to perform complicated procedures [biopsies, loop electrosurgical excision procedure (LEEP)]. Treatments for invasive CC (chemotherapy, radiation, radical hysterectomy) were referred to the highest tier referral hospitals. During this time, hospital operations across the country were limited or shut down due to multiple healthcare worker strikes (2019, Dec 2020–Feb 2021) and COVID-19 mitigation strategies (May–June 2020) affecting daily hospital operations and pilot study data collection. Data from enrolled participants were compared to data from historical controls who were screened for CC in the 6 months prior to CATSystem implementation April 2019–October 2019.

#### Study participants

All women ages 18–50 years who received CC screening in CCC or MCH during the study period were eligible. Due to the CATSystem’s use of SMS text messages, women also needed cell phone access to participate in the study. All participants provided written informed consent prior to study participation.

During the historical control period, paper CC screening registries from CCC and MCH were reviewed. Data were entered in an Excel spreadsheet by study staff. Although the registries followed the required ministry of health format, the data quality was highly variable regarding consistency, completeness, and evidence of follow up. Where paper records were incomplete, study staff followed up with providers to fill in gaps as much as was possible and ensured that duplicate records were reconciled; however, in many cases retrospective data remained incomplete.

### Study procedures

**FIGURE 2:**
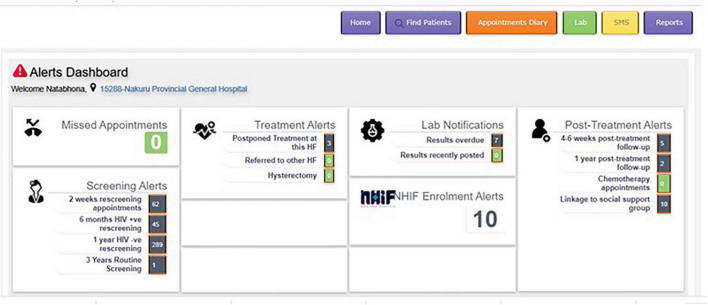
Cancer tracking system (CATSystem) dashboard.

Clinic providers or study staff informed women presenting for CC screening about the purpose of the CATSystem and asked eligible women if they would like to participate in the study or would prefer to receive standard of care (paper-based record keeping without action alerts or communication). Participants’ demographic and place/s of residence information were entered into the CATSystem upon enrollment. All subsequent counseling and clinical care data (including appointments, laboratory tests and results, treatment, rescreening) were entered into the CATSystem by a study research assistant. The CATSystem then used dates of services and other clinical criteria (i.e., screening results, appointment dates) to trigger electronic alerts to prompt providers when time-sensitive actions along the CC cascade of care were needed (see Figure 2 for an image of the dashboard). Clicking on each alert name would bring up a list of patients requiring that service, allowing providers to easily identify and initiate follow up among patients with incomplete services. Alerts were only resolved once the indicated action had taken place and had been recorded in the CATSystem. Finally, an informal “lunch and learn” style group discussion was held with providers (CCC Nurse, MCH Nurse, MCH Clinical Officer, two Gyno-oncologists and Laboratory technician) where facilitators and challenges to utilization and identified modifications to the CATSystem that could improve its utility and implementation.

#### Outcomes

The primary outcome of our study was “clinically appropriate care” after an abnormal CC screen defined as completion of any of the following actions: (1) Onsite treatment for precancerous lesions, (2) onsite or referred LEEP treatment for more severe precancerous lesions, (3) referral to a treatment center if suspected of invasive cancer, or (4) treatment of coinfections (e.g., cervicitis or STI), followed by a re-screen with appropriate follow-up within 3 months. We compared the pilot data to a 6-month retrospective record review of all female patients seen in settings where CC screening should have been conducted prior to CATSystem implementation.

#### Analyses

Descriptive statistics were calculated for demographic variables and risk factors for CC. Continuous variables were expressed as mean ± SD where applicable. Due to limitation in availability of historical control data, output is only available for intervention arm demographic and risk factor variables. The primary outcome was proportion of patients with a positive screening obtaining clinically appropriate care. A chi-square analysis was done on the categorical outcome to determine significance. Data was analyzed using SPSS v27.

## Results

A total of 491 patients were enrolled during the intervention period, and a total of 1,702 patient records were reviewed during the control period. Patient demographics are shown in Table 1. Due to lack of documentation in the CC registries, most demographic variables for control site participants were unavailable. The demographics collected through the CATSystem, however, indicate that the right target population were accessed during the pilot.

**TABLE 1:** Cancer tracking system (CATSystem) patient demographics.

Demographic	Intervention *N* = 491	Control *N* = 1,702
Age	38.6 ± 10.7 years	39.5 ± 11.3 years
**Education**		
Primary education	180 (40.9%)	–
Secondary education	150 (34.1%)	–
University/college	110 (25.0%)	–
**Weekly income**		
Less than 500 KES	196 (47.0%)	–
500–750 KES	64 (15.3%)	–
750–1,000 KES	36 (8.6%)	–
1,000–2,500 KES	121 (29.0%)	–
History of vulvar or cervical intraepithelial neoplasm	9 (1.8%)	–
First sexual encounter 18 years of age or earlier	196 (46.8%)	–
History of sexually transmitted infection	28 (5.7%)	–
HIV positive at baseline	128 (26.1%)	–

Among the 1,702 records screened in the control arm, 50 encounters had a documented positive screening test for CC, either VIA/VILI or PAP Smear or both. Among these 50 patients, 4 (8%) received further care in the form of clinically appropriate care that is, same day treatment by cryotherapy, or referral to a higher center for gynecology or oncology referral. Completion of referral treatment (i.e., surgery, radiation therapy, chemotherapy or other diagnostic or therapeutic interventions) could not be obtained, hence a documented referral for appropriate care was considered clinically appropriate follow up.

**FIGURE 3:**
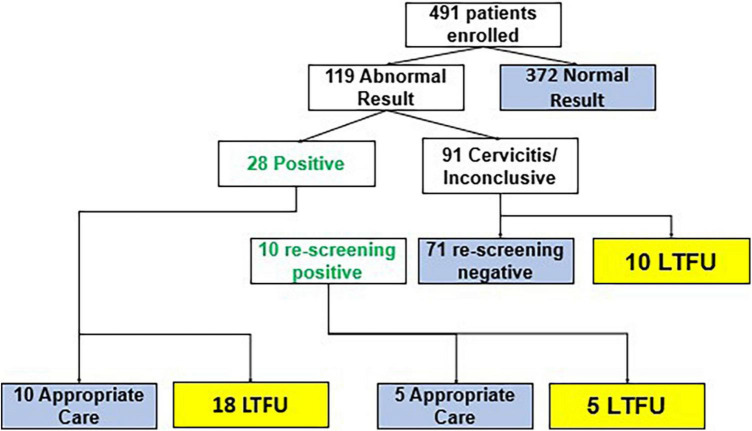
Flow of cancer tracking system (CATSystem) participants through cervical cancer care. Blue boxes indicate completion of care. Yellow boxes indicate loss to follow up prior to completion of care.

In the CATSystem arm, 119 patients had an initial abnormal screening result; of whom, 28 had a positive screen, of these, 10 received clinically appropriate care and 18 were lost to follow-up. Ninety-one patients had inconclusive results due to cervicitis and required rescreening. Of these 91, 10 were positive upon rescreening, 71 were negative, and 10 were lost to follow up. Thus, a total of **38** patients had a documented positive screening test. Of these 38, 15 (39.5%) received appropriate treatment that is same day treatment by cryotherapy, thermocoagulation or referral to higher center for further management (see Figure 3). In the CATSystem arm, patients continued to receive SMS reminders for re-visit for treatment or visit to a tertiary care facility for continuation of care. Ultimately, the CATSystem intervention led to a fivefold increase in the number of patients receiving clinically appropriate care (*p* < 0.01), after a positive screening result. That is, 15/38 (39.5%) compared to 4/50 (8%) among standard of care participants enrolled prior to CATSystem implementation received clinically appropriate care.

## Discussion

Preliminary evidence from this pilot study indicates that the CATSystem is a promising tool that can help clinicians improve rates of clinically appropriate treatment and follow up for suspected and invasive CC. The primary outcome of the study was the proportion of women with a positive screen result who received clinically appropriate care for precancerous lesions. With the CATSystem, we observed a fivefold improvement in receipt of clinically appropriate care after a positive screen: 15/38 (39.5%) among CATSystem participants compared to 4/50 (8%) among standard of care participants enrolled prior to CATSystem implementation. Despite these improvements, significant challenges in the provision of CC screening and care persisted at the system and patient level including: (1) Limited capacity of CC screening providers which meant missed screening, treatment and follow up opportunities, (2) a lack of clear linkage channels between screening and treatment points within the hospital, and (3) patient travel and treatment costs delaying their return for treatment. Informal conversations with providers confirmed the improvement in clinical care and reinforced many of the facilitators and challenges to utilization in the Kenyan context noted in the literature. Providers felt that the CATSystem features (dashboard alerts of missed screening and treatment appointments, pathology/lab results) gave them a more comprehensive picture of where their patients were in the cascade of care compared to the standard registers and allowed them to follow up with patients more easily.

Many of the system and patient-level challenges were outside the scope of the CATSystem and would need to be addressed at the system level through training and/or policy change. These challenges contributed to 61% of women not receiving clinically appropriate care, even with enhanced follow up through the CATSystem. While this is a drastic improvement from the 96% loss to follow up observed in the retrospective data, it highlights the urgent need for system and policy level changes to address remaining gaps. Such actions to improve could include: enhanced coverage for CC care through the National Hospital Insurance Fund (NHIF); training dedicated clinicians for CC screening and treatment services; prioritizing data and data systems, ensuring the availability of resources for CC screening and cryotherapy at all sub-county and higher hospitals; funding of more CC treatment centers; increased coordination between government, research, academic, and NGO stakeholders (28, 29).

Unlike in HIV, funding for CC prevention activities is not contingent on regular reporting, reducing the level of accountability. As such current data reporting levels are disjointed and incomplete contributing to the overall lack of monitoring data in Kenya. In pre-intervention data review of this study, the data quality was highly variable regarding consistency, completeness and follow up. Both CCC and MCH generally have one staff member tasked with carrying out the screening and record keeping, which on busy days affected data entry. Furthermore, for facility and county reporting purposes just noting the number of negative and positive screens is often adequate when reporting to county Ministries of Health. Once patients are referred, informal conversations with providers revealed that they are often too busy to follow up on the patient with other departments or the patient themselves resulting in a significant number of gaps in the paper registry. In addition, record overlaps were possible between registries as patients could be seen in both CCC and MCH departments. A way to fill in the gaps would have been to follow up with the patients, but due to limited staff and budget this was not possible. The need for improved data and data systems has been well noted (29, 30) and this is an area the National CC registry/board want to improve (28). In our study, the CATSystem significantly improved data quality and completeness and, indeed, was designed to help address this gap. Furthermore, the CATSystem was programmed with the potential to interact with the National Center Registry, an ability that to our knowledge no other CC eHealth technology in Kenya is currently able to do. Linking these systems could contribute to a comprehensive database of CC care and outcomes, nationally. Systems such as CATSystem can be integral to addressing the noted gaps and improving the CC cascade of care.

Early investments in screening can not only improve outcomes for women, but also reduce costs associated with later care and save lives. Public sectors costs for CC diagnostic and treatment procedures are estimated at $180.00 and $85.00–$1,500 (depending on stage), respectively (6). Studies report that only about 9–20% of CC patients have NHIF (National Health Insurance Fund) coverage, and this coverage is limited in what it provides, leaving patients to foot a substantial financial cost for treatment. At the main referral hospital KNH, patients can pay as much as $100–$300 for MRI’S and CT scans, and $300 per chemotherapy course, costs that are prohibitive given the national income average of $641 per month (31). Ultimately a multifaceted approach that includes the scale up of both screening and treatment, investment in data systems, and training is needed to address the burden of CC in Kenya (31). However, ensuring timely screening and rescreening through the less expensive VIA//VILI approach can ultimately lead to early diagnosis and treatment leading to reduced morbidity/mortality and preclude the need for more expensive procedures. The CATSystem can help systemize documentation and strengthen referral pathways; maximize the existing investments in CC care.

### Next steps

System-level and Intervention specific challenges, barriers/opportunities were identified in the pilot study that will be incorporated as we seek funding to conduct a rigorous evaluation (randomized control trial) of the system which will allow for longer follow up with more sites thus increasing the generalizability of findings. System level changes would include (1) working with facility, and local and national ministry of health to provide continuing education around CC screening, and (2) working with facility providers and administrators to develop clear in hospital linkage channels between screening and treatment points. Intervention-specific changes would include identifying specific personnel to enter data and based on informal conversations with providers the following additions to the system: (1) Sending SMS reminders not only to patients but to providers to remind when dashboard alerts come into the system, (2) sending post- operative SMS instructions after procedures such as colposcopy, LEEP and cryotherapy, (3) providing frequent supervision support and follow up training, and (4) setting up a data coordination system between involved departments. Finally, we will also include a cost analysis to assess the cost effectiveness of the CATSystem providing an argument for scaling up to other facilities.

### Limitations

The COVID-19 pandemic and the fall out within the health system including health worker strikes and limited access affected implementation of the study, this could in part account for the discrepancies we saw in numbers pre and post the intervention (32). However, we contend that the improvements are still noteworthy, especially given increased challenges with accessing healthcare during the intervention period due to COVID-19 restrictions. The incomplete CC paper registries due to limited staffing and busy providers as well as documentation errors made it difficult to collect full patient CC screening records for comparison with the system and we have no additional information on control participants outside of what has been reported. With more funding the work of following up with patients and other sources can be feasible. However, these limitations in data represent the standard of care in Kenyan facilities and emphasize the need for improved system of data management. Furthermore, the CATSystem is a passive system and can only reach the population presenting for care at study hospitals and this study only enrolled women presenting in MCH and CCC care. We acknowledge this as a limitation in that women who do not access care in these settings are represented in our study and our data do not allow us to estimate the proportion of women who may have been missed. We also do not have any additional information on the number of characteristics of women who were eligible for participation but chose not to participate. While the women missed by our study may have greater challenges accessing care and may bias our results, we have no information on their characteristics to expand upon this hypothesis. Rates of refusal will be more comprehensively documented in the proposed R01 and a plan to conduct community outreach to expand the reach of CATSystem has been drafted.

## Conclusion

There was a fivefold increase in the rate of clinically appropriate care after a positive screen among patients enrolled in the CATSystem, compared to pre-intervention data. Furthermore, it significantly improved data quality and completeness, which represents a priority among national stakeholders. Investing in systems to increase the rates of early detection and treatment of precancerious lesions can improve patients outcomes and ultimately reduce the costs associated with significantly more costly treatment of later-stage detection.

## Data availability statement

The datasets presented in this article are not readily available because data is identifiable. Requests to access the datasets should be directed to corresponding author.

## Ethics statement

The studies involving human participants were reviewed and approved by the University of Kansas Medical Human Research Protection Program. The patients/participants provided their written informed consent to participate in this study.

## Author contributions

All authors listed have made a substantial, direct, and intellectual contribution to the work, and approved it for publication.
